# Development of a web-based contact tracing and point-of-care-testing workflow for SARS-CoV-2 at a German University Hospital

**DOI:** 10.1186/s13756-021-00971-2

**Published:** 2021-07-02

**Authors:** Julian Zirbes, Christian M. Sterr, Marcus Steller, Laura Dapper, Claudia Nonnenmacher-Winter, Frank Günther

**Affiliations:** 1 Division of Infection Control and Hospital Epidemiology, Marburg University Hospital, Baldingerstrasse 1, 35043 Marburg, Germany; 2 Department of Infection Control, Hesse Health Office, Dillenburg, Germany; 3 Division of Information Technology, Marburg University Hospital, Marburg, Germany

**Keywords:** SARS-CoV-2, Contact tracing, Hospital environment, Digital tools

## Abstract

**Introduction:**

In late 2019, a novel coronavirus was detected in China. Supported by its respiratory transmissibility, even by people infected without symptomatic disease, this coronavirus soon began to rapidly spread worldwide.

**Background:**

Many countries have implemented different infection control and containment strategies due to ongoing community transmission. In this context, contact tracing as well as adequate testing and consequent quarantining of high-risk contacts play leading roles in containing the virus by interrupting infection chains. This approach is especially important in the hospital setting where contacts often cannot be avoided and physical distance is usually not possible. Furthermore, health care workers (HCWs) usually have contact with a variety of vulnerable people, making it essential to identify infections among hospital employees as soon as possible to interrupt the rapid spread of SARS-CoV-2 in the facility. Several electronic tools for contact tracing, such as specific software or mobile phone apps, are available for the public health sector. In contrast, contact tracing in hospitals often has to be carried out without helpful electronic tools, and an enormous amount of human resources is typically required.

**Aim:**

For rapid contact tracing and effective infection control and management measures for HCWs in hospitals, adapted technical solutions are needed.

**Methods:**

In this study, we report the development of our containment strategy to a web-based contact tracing and rapid point-of-care-testing workflow.

**Results/conclusion:**

Our workflow yielded efficient control of the rapidly evolving situation during the SARS-CoV-2 pandemic from May 2020 until January 2021 at a German University Hospital.

## Introduction

In late 2019, a novel coronavirus (SARS-CoV-2) was detected in China. After a rapid spread throughout the world, the World Health Organization (WHO) declared it a pandemic on the 11th of March 2020 [1].

## Background

SARS-CoV-2 is primarily transmitted through the respiratory intake of viral particles that are excreted by coughing, speaking or breathing [2, 3]. Literature shows transmission can happen even before symptoms occur [4–6]. Infection from contaminated surfaces is also discussed [7–9]. Due to ongoing community transmission, many countries have implemented infection control measures following a containment strategy [1, 10]. For containment, contact tracing and an adequate testing strategy are essential [11]. In the early stages of the pandemic, contact tracing helped confine outbreaks in Singapore and China by identifying infected people before they showed symptoms [12, 13]. Reports suggest that the earlier contact tracing and quarantining are performed, the better an outbreak can be controlled [14]. Contact tracing begins with identifying and listing people who were in contact with an infected person. Contacts are then informed about possible transmission and resulting infection control measures (quarantine/self-isolation, symptom journals, planned testing). These conventional methods involve the need for great personal resources [1, 15]. Facing rising numbers of infections, a variety of countries and organizations began developing technological tools and informational technology (IT) solutions to complement conventional contact tracing in the public health sector [1, 16–18]. Also, many countries have introduced mobile phone apps for contact tracing [19, 20] but have faced concerns about data safety and privacy as well as possible limited use by age groups who are not accustomed to newer technologies [1, 16, 19]. Overall, there are multiple recommendations by public health organizations to perform contact tracing with IT support [1, 2, 11, 21]. However, these are only partially applicable to hospitals, where contacts often cannot be avoided and physical distance cannot be kept. Many vulnerable groups are concentrated in relatively small spaces. Additionally, only a few HCWs care for dozens of patients regularly. All of this makes it particularly important to break chains of infection by identifying infected employees quickly and efficiently [22]. Combining contact tracing, adequate testing and the fast initiation of infection control measures can actively prevent the spread of infections, especially in hospitals [23]. In this study, we report the development of our containment strategy to a web-based contact tracing and rapid point-of-care-testing (POCT) workflow, yielding in efficient control of the rapidly evolving situation during the SARS-CoV-2 pandemic from May 2020 until January 2021 at a German University Hospital.

## Methods

### Phase 1: Paper-based workflow—March 2020

The first contact tracing of SARS-CoV-2 at Marburg University Hospital was performed in March 2020 only weeks after the first confirmed positive cases had appeared in Germany. Initially, the division of infection control was provided with handwritten lists of telephone numbers belonging to HCWs who probably had contact with detected index cases. ICPs then had to chase reported contacts on the phone to identify further contacts and assess their individual risk of developing SARS-CoV-2 infections. Contacts were advised to get a PCR-test by a nasopharyngeal swab on day 1 and day 5. Depending on contact intensity they were told to preventively self-isolate at home and wait for to the local health authorities to contact them and to evaluate the need for quarantine. The results of the PCR tests had to be actively pursued by the ICP through the laboratory information system (LIS). Contact patients were identified separately using the hospital information system (HIS). This very time consuming process, which was primarily manually oriented, quickly reached its maximum possible capacity in the course of the SARS-CoV-2 pandemic in early 2020.

### Phase 2: Computer-based workflow—April 2020

In order to accelerate the workflow, we implemented a standardized paper-based contact form that could be downloaded from the hospital intranet. Contacts among HCWs had to identify themselves autonomously, fill out the form and send it to the division of infection control. Contacts among patients still had to be identified separately using the HIS. Unfavorably, forms also reached the division of infection control in multiple ways, making it necessary to further standardize the input and transfer it for the electronic processing of contact lists. Therefore, this workflow might have omitted the time-costly, telephone-based retrieval of contacts but contacts still had to be informed and instructed separately. Soon, it became apparent that this computer-based workflow would not be feasible in a phase of higher emergence of infections due to a shortage of IT support and standardization. Altogether the limitations of the computer-based workflow led to the development of an intranet-based workflow (Phase 3) that was implemented in May 2020.

### Phase 3: Intranet-based workflow—May 2020

**Fig. 1:**
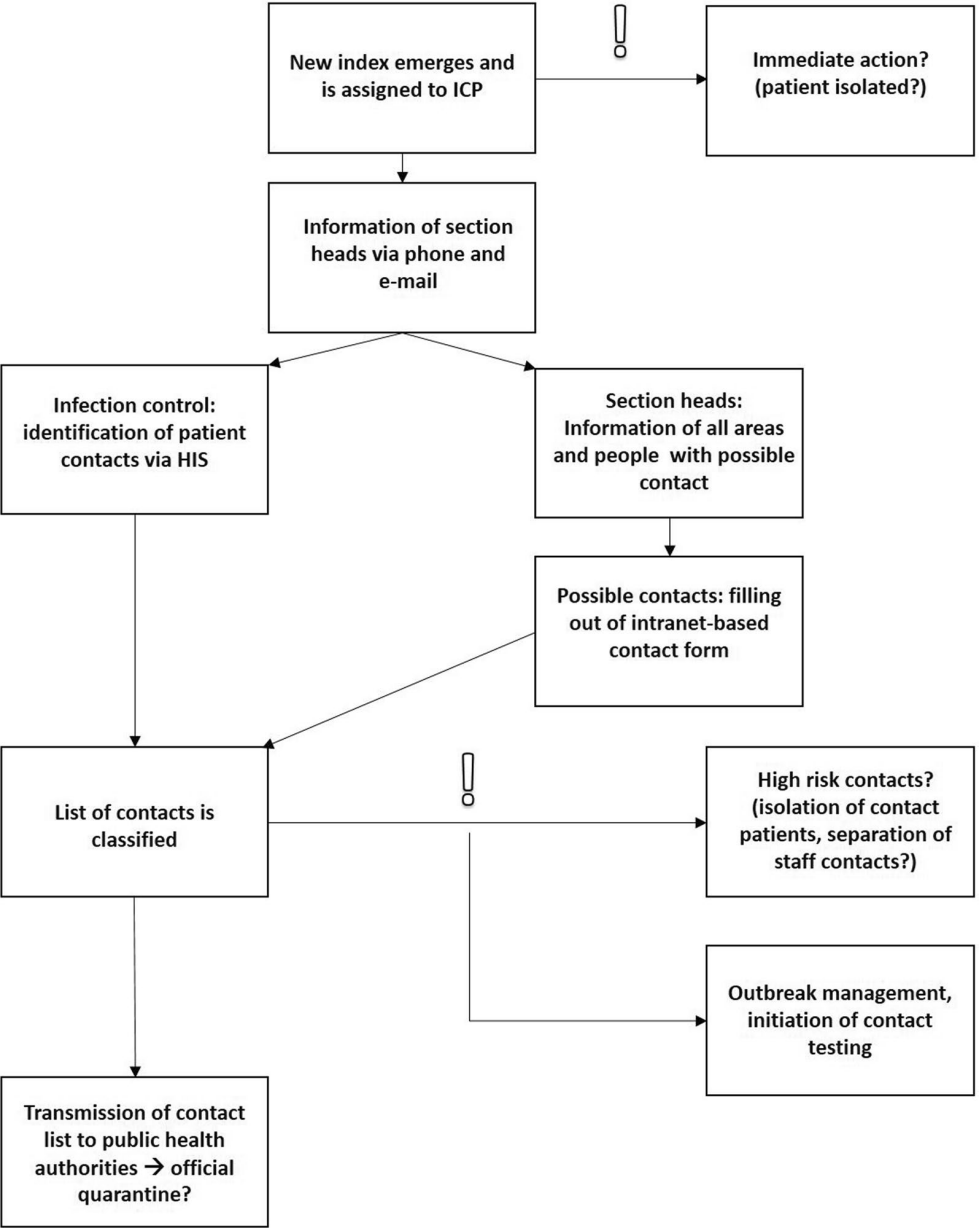
Contact tracing workflow

As soon as a new SARS-CoV-2 index case was reported, it was assigned to an ICP. To ensure efficient contact tracing, the ICPs proceeded according to a standard operating procedure (Fig. 1). The ICP called the direct supervisor or the head nurse of the affected section to inform them about the new index case and planned infection control measures including screening regime. Additionally, a standardized e-mail was sent to each affected unit containing information about the newly detected index case, the necessary infection control measures and a link to the intranet-based contact form. Attached to this email, a list with all different occupational groups and functional areas of the hospital is sent to the recipients to subsequently identify and inform incorrectly and not-yet identified contact persons and groups.

The intranet based workflow comprised a form with checkboxes for index case, contact duration, kept distance, worn PPE (e.g. face mask, respirator), and input boxes for personal data and existing symptoms. As soon as the relevant information was submitted, the ICP-team received an e-mail notification for every new contact. The case-ICP was then able to access all necessary information in the form of a table listing all contacts among employees for a specified index case.

High risk contacts (e.g. symptomatic people, or people with long close contact without wearing PPE) were automatically highlighted by an algorithm to accelerate proceedings. Thus, ICPs could rapidly identify high risk contacts and act consequently (e.g. isolation of contact patients, separation of staff contacts).

**Fig. 2:**
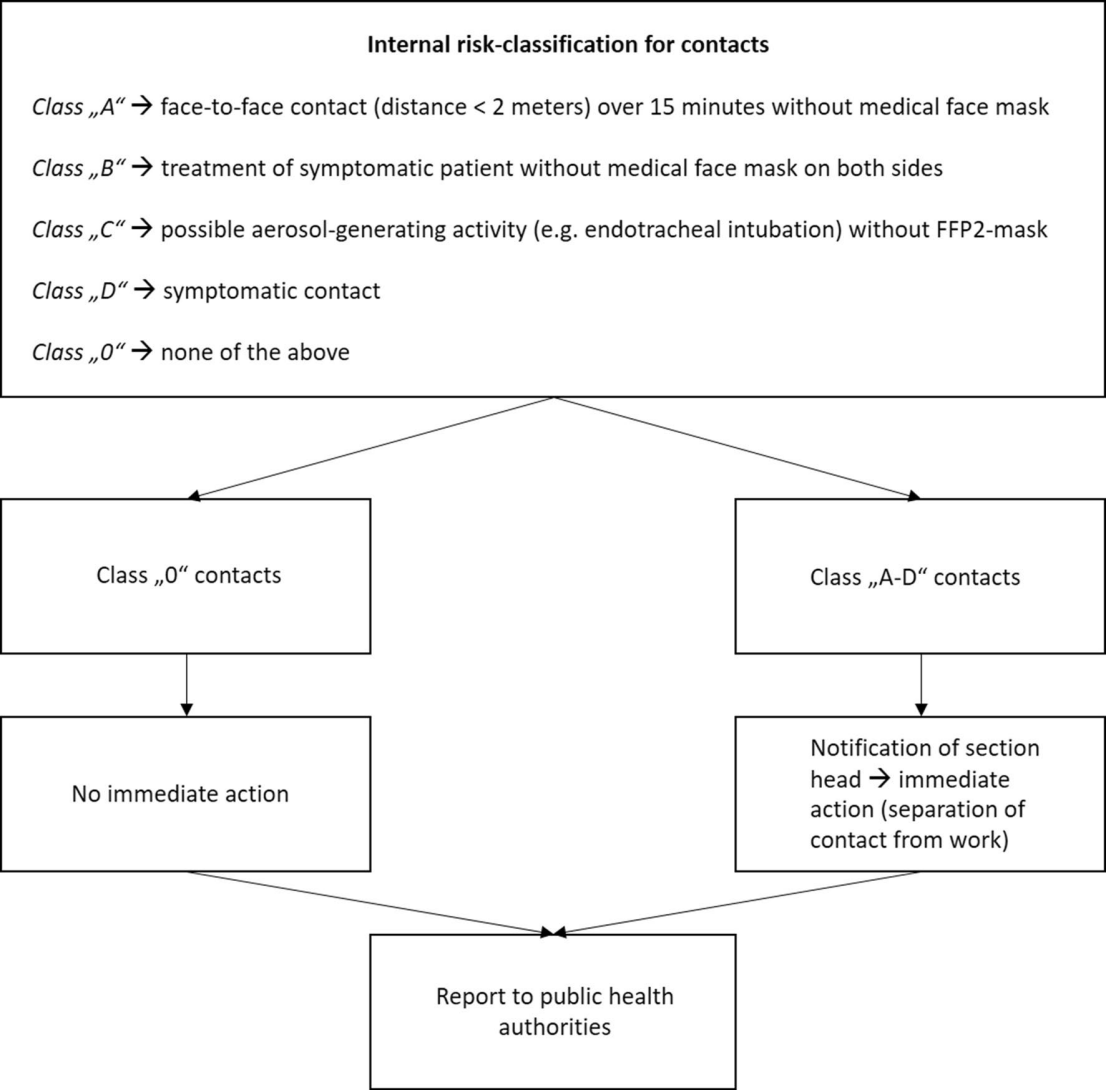
Internal risk-classification of contacts

Other contacts were evaluated according to our internal risk classification (Fig. 2) which was based on the recommendations of the Robert-Koch-Institute (RKI) but designed more straightforward in order to consider the special circumstances of a university hospital [24] (Fig. 2). Additionally, ICPs had to answer to following questions, to take appropriate infection control measures depending on the individual risk assessment:

- Is the new index a patient or a hospital employee?
- If it is a patient, is he/she still admitted? If yes, since when? Is he or she isolated?
- If it is an employee, is he/she working at the moment? If yes, in which clinic or section?
- Is the index symptomatic? If yes, since when?
- Do we have preexisting test results (PCR, antigen test)? If yes, what and from when are they? How are ct value and ct course?

Contacts among patients were traced separately using the HIS before they were added to the contact list. In Germany, it is legally obligated, to provide the local public health authorities with personal data of close contacts. To ensure data protection requirements, the generated lists were uploaded to a protected cloud system which was operated by the local authorities.

### Phase 4: Intranet-based test regime—October 2020

Once rapid POCTs were available in Germany, the tests became part of our contact tracing and testing procedure. Our objective was to decrease time to result and increase testing capacity. In order to control a possible viral spread, it is crucial to get results as soon as possible after testing. Hence hospital employees were tested at least three times every 48 h after last contact to the index case by using SARS-CoV-2 Rapid Antigen Test (Roche, Basel, Switzerland). For contacts among patients or symptomatic contacts the same schedule was applied, but testing was performed via PCR. Additionally, positive rapid POCTs had to be confirmed by PCR. To ensure data protection, an anonymous barcode with a personal-ID-number was generated for every HCW who filled out the intranet-based contact form. Without a barcode, HCWs couldn’t get tested at the clinic’s test center. POC screening results were automatically submitted via the intranet-based tool and displayed in the contact tracing list. PCR results had to be reviewed manually as they were not linked to the contact tracing tool. Results were reviewed by the case-ICPs on a regular basis. This way, it also came to our attention if a specific employee had not yet used their barcode for testing. In addition, the tested employees were able to retrieve their test results by entering their ID-number into the intranet tool. The tool provided the division of infection control with all important information at any time by directly linking the POCT results to a contact person while also having information about index case and the individual risk assessment available. It therefore facilitates rapid identification of positive contacts among employees, continuous evaluation of the ongoing infection process and assessment of the efficacy of outbreak control measures.

To keep the threshold for hospital staff to be tested as low as possible, a second web-based form for voluntary SARS-CoV-2 testing was implemented in October 2020. Using this form enables HCWs to generate barcodes for testing without having been in contact with a SARS-CoV-2 case in the hospital setting or without any other reason. This form facilitates testing for various private reasons and for intermittent routine tests, for example. The tracing lists are similar to those used for contact tracing, but they do not include data on index cases or contact data and are followed up on separately by the ICP.

Personalized data from the web-based contact tracing was accessible for the IPCs only until 14 days after entry using a password-protected interface. HCWs can call up their individual screening results anonymously for an unlimited period of time via a query mask in the hospital intranet by using an individualized code they had received after entry in the contact tracing mask.

Only non-personalized data on the number of contacts, the duration of the contact, the time of the contact and the date of test results were used for retrospective analysis of the POCT workflow effectivity and in purpose of this report. The statistical analysis of our data was performed using SPSS (IBM, Armonk, USA).

## Results

**Fig. 3:**
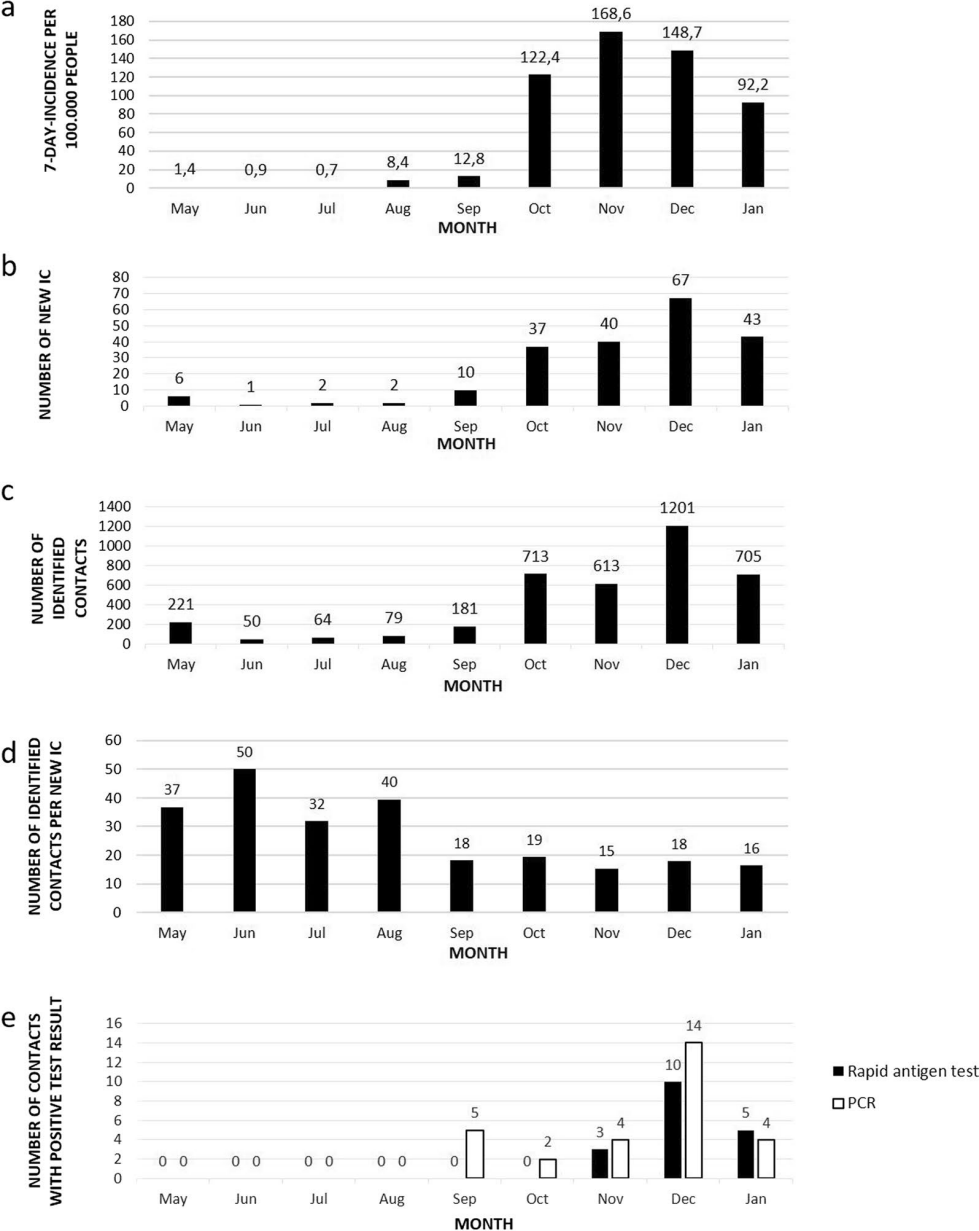
Courses of local incidence, new index cases and identified contacts. **a** Local SARS-CoV-2 7-days-incidence per 100.000 people [25]. **b** Number of new SARS-CoV-2 index cases (IC) per month 2020/21. **c** Number of identified contacts per month 2020/21. **d** Number of identified contacts per new index case (IC) per month 2020/21. **e** Number of contacts among employees tested positive for SARS-CoV-2 per month 2020/21

Between May and September 2020, 21 index cases were detected and traced in Marburg University Hospital. A total of 595 contacts were identified among hospital employees (Fig. 3). The conducted contact tracing procedures revealed five new SARS-CoV-2 infections among these contacts in our hospital.

**Fig. 4:**
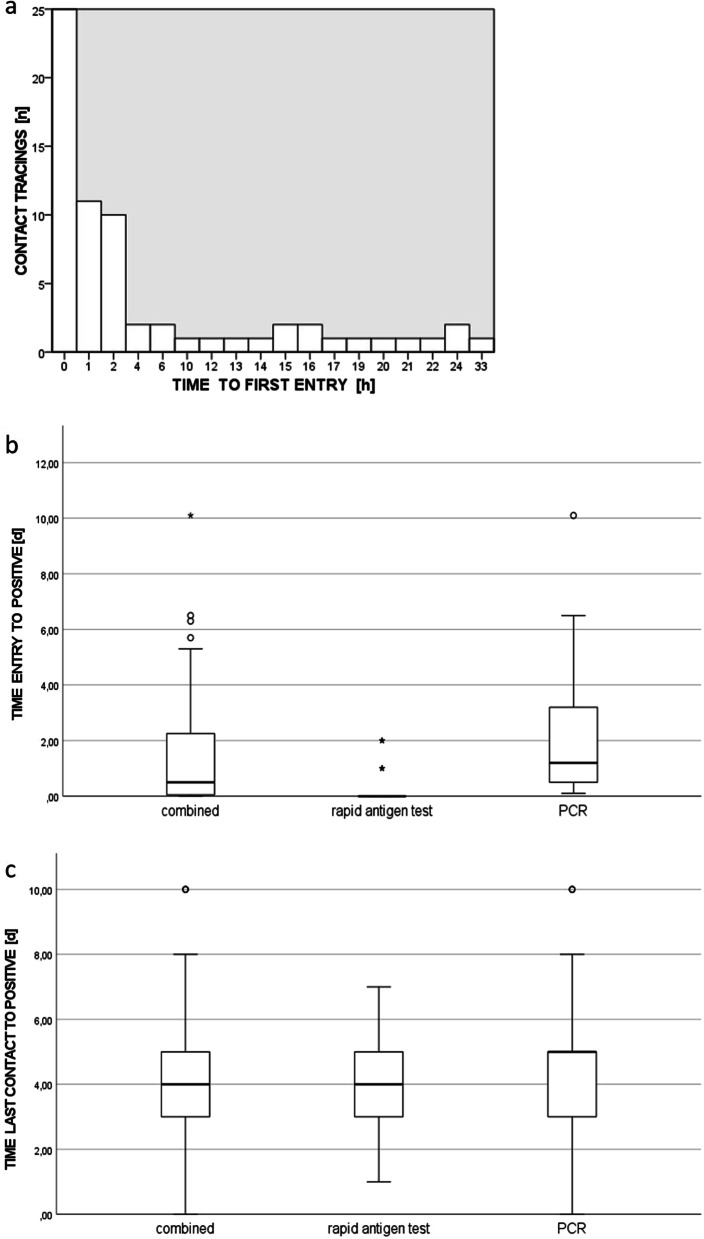
Time-span evaluation. **a** Time initial e-mail to first contact form entry. **b** Time last index contact to positive test result. **c** Time contact form entry to positive test result

Between October 2020 and January 2021, 187 new index cases with a total of 3232 resulting contacts among hospital employees were registered (Fig. 3). This represented a nearly tenfold increase in monthly traced contacts compared to the summer months (May to September: 90; October to January: 808). The number of new index cases matched the local SARS-CoV-2 incidence at the time [25] (Fig. 3). The number of contacts per index case remained high during the summer months and reached a lower and stable level in winter (Fig. 3). The time between notifying the affected sector via e-mail and reception of the first related contact form is depicted in Fig. 4. The mean time to first entry was 5.2 ± 8 h. Fifty-nine percent of the first entries were received even before the initial e-mail had been sent.

Environmental testing, according to the contact tracing regime implemented, revealed 24 newly SARS-CoV-2-infected employees (Fig. 3), most of which (19 out of 24) tested positive within one week after their last index contact. Three of the remaining five people received their first documented in-house testing over two weeks after their last index contact and were excluded from the time-span evaluation. The average time between the last contact to the index case and a positive test result was 4 ± 1.7 days for rapid antigen tests and 4.5 ± 2.4 days for PCR (p > 0.05) (Fig. 4). Five contacts only received PCR testing, while one person had a false-positive rapid antigen test that could not be confirmed by PCR. The average time between contact list entry and a positive test result was 0.3 ± 0.7 days for rapid antigen tests and 2.1 ± 2.3 days for PCR (p < 0.05) (Fig. 4). A total of 78.9% of positive rapid antigen tests were conducted within one day of the contact form reaching the infection control department.

In addition, 579 employees were registered using the intranet form for voluntary and routine testing from October 2020 until January. In this context, 8 new index cases were identified.

## Discussion

In this study, we describe the development of our containment strategy to a web-based contact tracing and POCT workflow. This workflow provided fast test results necessary to handle the rapidly evolving situation during the SARS-CoV-2 pandemic and enables structured and standardized contact tracing.

Chasing reported contacts on the phone, evaluating the risk of transmission individually and tracing test results in the LIS was very time consuming. We therefore developed a standardized contact form in April 2020, which was made available for download from the hospital intranet. Because of a shortage of standardization, we subsequently designed an intranet-based contact tracing tool with low threshold and good accessibility in May 2020 to provide our ICPs with easily available and structured contact data. Thus, most contact forms were received within only hours (Fig. 4). Sometimes, the forms were filled out even before the initial e-mail had been sent. Consequently, infection and eventually outbreak control measures could be implemented quickly.

High risk contacts were automatically highlighted in structured lists by an algorithm for further accelerating the process. Filtering out those contacts was very important, since temporarily public health authorities were only able to contact these people with considerable delay due to high work load. This time gain is of utmost importance for infection control as it limits spreading of the virus in the hospital setting [23].

To perform efficient contact tracing, cases were assigned to specific ICPs who had to follow a SOP. Affected sections were involved from the very beginning by directly informing those responsible about our measurements (e.g. infection control measures, screening intervals) via telephone and e-mail. Addressing of section heads managed and divided responsibilities in a clear and documented way. In contrast to early 2020, all relevant sections and function areas were systematically queried, leading to comprehensive acquisition of contacts.

Soon after the local incidence had started to increase in October 2020 (Fig. 3), in a final adjustment, POC-testing with integration of POCT results in the contact lists became part of our workflow. Thereby, we were able to handle as many as 1,201 contact cases in December 2020 with temporarily only 3 ICPs on duty. Even more importantly, we could further accelerate our workflow. Almost 80% of positive rapid antigen tests were conducted on the same day the person filled out their contact form, preventing further delay until PCR results became available (Fig. 4). Both rapid antigen- and PCR-tests identified the majority of infected contacts within four to five days after their last index contact (Fig. 4). Consequently, the final adjustment provided an additional acceleration of our process. In addition, following up screening results and identification of subsequent cases was simplified.

In terms of a potential risk for others, there are huge differences between patients and HCWs. HCWs do have more contacts than patients. Therefore, HCWs can spread the virus throughout the hospital more easily. Moreover, the workforce of HCWs is needed most in a pandemic situation. Thus, we decided to test HCWs with POCT immediately after contact to get a fast test result combined with a high testing capacity to a reasonable price. In doing so, we were able to ensure patient care with low risk of viral spread at the same time. Patients on the other hand, were solely tested by PCR—ensuring highest possible sensitivity. The lower sensitivity of POCT compared to PCR testing was counterbalanced by repeated point-of-care- testing of our employees [26]. To rule out false positives and increase specifity we performed PCR confirmation of positive POCT.

Anonymization (barcode with personal-ID-number) provided sufficient data protection. Employees were able to get tested anonymously and retrieve their test results by entering their ID-number into the intranet tool.

The structured lists lead to the delay-free submission of contacts, which have already been filtered by relevance internally, to public health authorities, reducing their incoming data amounts.

However, the evaluation of time spans is complicated by HCWs who do not adhere to given screening intervals, as well as people getting tested externally or anonymously and making first use of their testing barcode after quarantine has ended.

The extensive usage of the additional form for voluntary and routine testing confirms its low threshold. A low threshold for voluntary and routine testing is required, as it enables the early identification of new index cases of non-nosocomial infections and the rapid implementation of infection prevention measures [22]. In our case, 8 new non-nosocomial infections were identified. This also underlines the importance of automated test result feedback to the infection control departments.

The relatively high numbers of new index cases compared to local incidence in September 2020 (Fig. 3) could be attributed to an outbreak, which included four out of five index cases in September. A change in problem perception and further tightening of infection control measures might be responsible for the reduction of identified contacts per new index case afterwards (Fig. 3). Therefore, this example underlines how data from contact tracing can help optimize internal procedures and adherence to infection control measures.

Limitations of the process include the dependence on the quality of data entered in the form. Implausible entries were still followed up on via telephone and corrected individually. In this context, further optimization and simplification of the system and entry mask is necessary in the future. In summary, contact tracing systems must be continuously adapted to the requirements and developments of the pandemic.

## Conclusion

In conclusion, our web-based contact tracing and POCT workflow for SARS-CoV-2 offered fast test results as well as structured and comprehensive contact tracing for hospital employees. Using this workflow, we were able to handle the increased number of index cases during the rapidly evolving pandemic between October 2020 and January 2021. Furthermore, our data provide evidence that using frequent intermittent rapid antigen testing for follow-up of contacts is effective for the identification of infected contacts, therefore preventing the spread of infections in the hospital setting. In order to manage rapidly changing situations like future pandemics, further development of technical solutions and POC-testing for contact tracing hospitals is needed.

## Data Availability

The datasets used and/or analysed during the current study are available from the corresponding author on reasonable request.

## Abbreviations

CDC: Centers for Disease Control and Prevention
ECDC: European Centre for Disease Prevention and Control
HCWs: Health care workers
HIS: Hospital information system
HMSI: Hessian Ministry for Social Affairs and Integration
IC: Index case
ICPs: Infection control practitioners
IT: Informational technology
LIS: Laboratory information system
POCT: Point-of-care-test
RKI: Robert-Koch-Institut
WHO: World Health Organization
